# Placental-Derived Mesenchymal Stem Cells Triggers Lipid Metabolism in a Rat Model Thioacetamide-Induced Ovarian Disease via Increased CPT1A Expression for Mitochondrial Dynamics

**DOI:** 10.3390/cells14241932

**Published:** 2025-12-05

**Authors:** Hyeri Park, Jun Hyeong You, Jin Seok, Dae Hyun Lee, Hankyu Lee, Gi Jin Kim

**Affiliations:** 1 Department of Biomedical Science, CHA University, Seongnam-si 13488, Republic of Korea; hyeyeyeri@chauniv.ac.kr (H.P.); yjh950210@gmail.com (J.H.Y.); ldh1532@chauniv.ac.kr (D.H.L.); hglee@plabiologics.com (H.L.); 2 Research Institute of Placental Science, CHA University, Seongnam-si 13488, Republic of Korea; 3 Asan Institute for Life Sciences, Asan Medical Center, Seoul 05505, Republic of Korea; jjin8977@amc.seoul.kr; 4 Advanced PLAB, PLABiologics Co., Ltd., Seongnam-si 13522, Republic of Korea

**Keywords:** placenta-derived mesenchymal stem cell, ovarian dysfunction, lipid metabolism, mitochondria dynamics, steroidogenesis

## Abstract

Lipid accumulation disrupts mitochondrial dynamics, leading to dysfunctional energy metabolism and increased oxidative stress. However, the relationship between mitochondrial dynamics and ovarian function in therapeutic contexts is still not fully elucidated. Therefore, the objective of this study was to demonstrate whether increased carnitine palmitoyltransferase 1A (CPT1A) expression induced by placenta-derived mesenchymal stem cells (PD-MSCs) improves ovarian function in ovaries of a lipid toxicity-induced rat model by regulating lipid metabolism and mitochondrial dynamics. A rat model of injury was induced through intraperitoneal administration of thioacetamide (TAA) for 12 weeks. During the 8th week of induction, PD-MSCs (2 × 10^6^ cells) were transplanted via the tail vein. Initially, we examined the engraftment of PD-MSCs. The inflammatory response (e.g., IL-6, TNFα) and apoptosis (e.g., LDH levels, TUNEL assay) were significantly increased in the non-transplanted (NTx) group compared to the normal group; however, they were significantly decreased in the transplanted (Tx) group compared to the NTx group (* *p* < 0.05). Additionally, oxidative stress was attenuated through the regulation of mitochondrial dynamics, including the expression of DRP1, ATP5B, and PGC1α, in the Tx group compared to the NTx group (* *p* < 0.05). In the NTx group, abnormally accumulated lipid droplets were observed due to dysfunctional mitochondria, whereas in the Tx group, the accumulation of lipid droplets and the expression of CPT1A were significantly comparable to those in the normal group (* *p* < 0.05). The levels of the steroidogenesis markers (e.g., CYP11A1 and HSD3β1) were decreased in the NTx group compared to the normal group and increased in the Tx group compared to the NTx group (* *p* < 0.05). The levels of sex hormone and follicular development were protected in the Tx group compared to the NTx group. Furthermore, cocultivation of PD-MSCs with etomoxir (CPT1A inhibitor)-treated primary theca cells increased the expression of steroidogenesis. In conclusion, PD-MSCs improve ovarian function in TAA-induced injury by reducing lipid accumulation and oxidative stress through the regulation of lipid metabolism and mitochondrial dynamics. The upregulation of CPT1A and related mitochondrial proteins contributes to enhanced steroidogenesis and restoration of ovarian homeostasis. These findings offer new insights into the application of stem cell therapies for reproductive medicine.

## 1. Introduction

Steroid production in the ovaries is a key process where ovarian cells generate hormones essential for maintaining reproductive tissue, regulating ovarian function and ovulation, and establishing pregnancy [1]. Through follicular development, FSH and LH stimulate the production of cyclic adenosine monophosphate (cAMP) in granulosa cells and theca cells and, through the same mechanism, induce growth and estrogen secretion [2]. Steroidogenesis in ovaries is achieved by the interaction between granulosa and theca cells. The follicle-stimulating hormone (FSH) on granulosa cells induces luteinizing hormone (LH) receptor expression and increases steroidogenesis. The LH on theca cells works synergistically with FSH to act on FSH-stimulated follicles, maintaining their growth and ultimately facilitating the processes of luteinization and ovulation [3,4]. In particular, theca cells initiate follicular steroidogenesis and are crucial for reproductive function by supplying the androgen substrates necessary for ovarian estrogen synthesis [5].

Steroidogenesis is linked to lipid metabolism, and the cholesterol used for this process is converted into steroids necessary for ovarian follicle maturation [6]. In the absence of steroidogenesis, excessive lipids cause lipotoxicity and reproductive dysfunction [7,8]. Cholesterol is derived from lipid droplets; however, excessive lipid accumulation induces lipotoxicity, mitochondrial dysfunction, and oxidative stress in the ovaries [9]. Thus, abnormal lipid accumulation results in a similar phenotype as menopause [10]. Moreover, various adipokines regulate ovarian steroidogenesis, so excessive lipid accumulation leads to an overproduction of androgens, which in turn causes polycystic ovary syndrome (PCOS) [11]. Furthermore, lipid accumulation was associated with mitochondrial dysfunction [12].

Mitochondria are major keys to generating ATP through cellular respiration and regulating various metabolic processes as the powerhouses of the cell. However, when mitochondrial function is impaired, access to stored fuels such as glycogen and lipid droplets is disrupted [13]. Accumulation of fatty acids around mitochondria contributes to lipid peroxidation through reactive oxygen species (ROS) [14]. Igosheva and their colleagues demonstrated that oocytes from obese mice exposed to a high-fat diet not only exhibited abnormal morphology compared to oocytes from normal mice but also had lower ATP levels and higher ROS levels [15]. ROS within the ovaries affect the progression of meiosis II, reduce gonadotropin secretion and DNA damage, and inhibit ATP production. This is directly associated with decreased oocyte maturation and fertilization rates, lower embryo quality, and reduced pregnancy rates [16]. Ding and their colleagues suggested that mtDNA mutations can cause mitochondrial dysfunction and may be involved in the development of insulin resistance (IR) in PCOS [17].

CPT1A is a rate-limiting enzyme in fatty acid oxidation (FAO), responsible for transferring long-chain acyl groups from acyl-CoA esters to carnitine. It facilitates the transport of fatty acids into mitochondria for β-oxidation, a process essential for ATP production [18,19]. By regulating this key step, CPT1A prevents lipid accumulation, maintains lipid homeostasis, and contributes to the prevention and treatment of obesity and metabolic syndrome-related disorders [20]. According to Ren and their colleagues, they demonstrated that CPT1A expression was activated in the liver of high-fat diet mice [21]. Tepavčević S and their colleagues demonstrated that the expression of CPT1A was increased in the heart of a dihydrotestosterone (DHT)-induced rat model [22]. Such studies suggest that in lipid metabolism disorders, mitochondrial activation for lipid reduction leads to increased expression of CPT1A [23]. Furthermore, CPT1A plays a crucial role not only in lipid metabolism but also in the maturation and quality of oocytes through intracellular lipid storage. Sanchez L and colleagues demonstrated that inhibiting FAO with the CPT1 inhibitor etomoxir during IVM impairs meiotic maturation in bovine [24].

Moreover, lipotoxicity is well known to cause mitochondrial dysfunction by inducing ROS [25,26,27]. This lipid peroxidation directly damages phospholipids and can also cause programmed cell death. Recently, it was reported that thioacetamide (TAA) induces an inflammatory response and oxidative stress in the ovaries and causes similar symptoms, such as estrogen deficiency, in a model of ovarian dysfunction generated by an ovariectomized rat model [28]. TAA is known to induce lipid accumulation and oxidative stress in the liver and is widely used to determine the mechanism of lipid metabolism dysfunction in various diseases [29]. However, it has not yet been reported whether TAA affects steroidogenesis and thus ovarian function or mitochondrial dysfunction by promoting abnormal lipid accumulation in the ovaries.

To date, a range of therapeutic approaches, including hormone replacement therapy (HRT), assisted reproductive technology (ART), and immunomodulation therapy, have been applied to treat ovarian diseases, but effective treatments for ovarian diseases resulting from excessive lipid accumulation and abnormal lipid metabolism, especially PCOS, an ovarian disease caused by metabolic abnormalities, are lacking [30]. Metformin, an insulin sensitizer, exerts its effects by decreasing hepatic glucose production, reducing intestinal glucose absorption, and enhancing cellular glucose utilization. In women with PCOS, these mechanisms can lead to improved menstrual cyclicity, restored ovulation, and reduced androgen levels; however, it may also cause side effects such as gastric distress and vitamin B12 deficiency. Additionally, it has temporary effects and lacks a clear mechanism for improving follicular development [31,32]. ART is a medical intervention aimed at fertilizing oocytes and sperm in vitro or transferring embryos, rather than improving the quality or function of oocytes. Therefore, for managing the high miscarriage rate in patients with PCOS, ART should be accompanied by the management of metabolic and hormonal factors [33,34]. GLP-1 receptor agonists, which exhibit immunomodulatory effects, have been shown to reduce insulin resistance and regulate hormonal imbalance in patients with PCOS, thereby promoting regular menstrual cycles; however, there have been reports of treatment discontinuation due to the burden of subcutaneous administration and the high cost of the medication [35,36].

As a result, stem cell therapy has attracted attention as a next-generation treatment that can overcome the limitations of treatments for intractable diseases [37]. The advantages of stem cells include higher self-renewal potential, multi-differentiation potential, and immunomodulatory activity [38]. Regarding the therapeutic efficacy of mesenchymal stem cells (MSCs) in animal models, a previous study reported that intraovarian transplantation of MSCs can alleviate multiple metabolic abnormalities associated with PCOS that have been observed in a mouse model of the disease [39]. 

Human placenta-derived mesenchymal stem cells (PD-MSCs) are valuable because, unlike other stem cells, they pose no ethical concerns regarding isolation, as they are obtained from discarded placental tissues. In particular, PD-MSC transplantation has been reported to restore mitochondrial function, promote FAO, and reduce lipid accumulation in hepatocytes [28]. In our previous study, PD-MSCs were found to increase autophagy and decrease apoptosis in a liver disease model [40]. Additionally, we confirmed that PD-MSCs improve ovarian function by exerting antioxidant and vascular remodeling effects in an ovarian failure model [41,42]. However, whether PD-MSCs can regulate lipid metabolism and improve mitochondrial function in an ovarian failure model has not yet been reported. Therefore, in this study, we investigated whether PD-MSC transplantation affects lipid metabolism in ovarian tissue after TAA-induced lipid toxicity and how it alters follicular development and function through lipid metabolism via regulation of mitochondrial CPT1 expression in TAA-injured rat ovarian tissue.

## 2. Materials and Methods

### 2.1. Establishment of TAA-Induced Ovarian Injury Model

Seven-week-old female Sprague–Dawley rats (Orient Corporation, Seongnam, Republic of Korea) were housed under specific-pathogen-free (SPF) conditions (21 ± 2 °C, 50 ± 10% humidity, 12 h light/dark cycle) with unrestricted access to food and water. After one week of acclimatization, animals were randomly assigned to three experimental groups: (1) untreated controls, (2) thioacetamide-treated non-transplantation (NTx) rats, and (3) thioacetamide-treated rats receiving PD-MSC transplantation (Tx). Chronic ovarian damage was induced through intraperitoneal injection of thioacetamide (TAA; 150 mg/kg, Sigma–Aldrich, St. Louis, MO, USA) twice weekly for 12 weeks. All procedures were conducted following approval from the Institutional Animal Care and Use Committee of CHA Laboratory Animal Research Center (IACUC No. 200033). Rats were inspected daily, with increased observation frequency during dosing. Animals showing >15% body-weight reduction or severe distress were humanely euthanized by isoflurane anesthesia followed by a secondary method. No animals or data points were excluded except in cases meeting these criteria.

### 2.2. Stem Cell Culture and Transplantation into a Rat Model of TAA-Induced Injury

Human placental tissues collected at term (≥37 weeks) with informed consent were used under the approval of the CHA Gangnam Medical Center IRB (IRB-07-18). PD-MSCs were isolated and expanded under conditions previously validated in our laboratory [43,44]. After eight weeks of TAA exposure, animals were anesthetized with avertin prior to transplantation. Cultured PD-MSCs at passage 13 were labeled with PKH67 (Sigma–Aldrich) and delivered intravenously via the tail vein (2 × 10^6^ cells suspended in 200 µL DPBS). Animals in the NTx group received the same volume of DPBS. Four weeks later, animals were euthanized by CO_2_ inhalation, and blood and ovarian tissues were harvested for subsequent molecular and histological analyses. No unexpected adverse events were observed during the study. Animals were monitored daily for general health, and only minor, transient changes in body weight were observed, which were considered expected outcomes under the experimental procedures.

### 2.3. Primary Theca Cell Isolation from Rat Follicles and Cell Culture

Ovaries from healthy 7-week-old female rats were excised aseptically following CO_2_ euthanasia. After removing adherent fat, follicles were punctured with a 26 G needle in McCoy’s 5A medium (Gibco (Thermo Fisher Scientific, Waltham, MA, USA)) supplemented with 2 mM L-glutamine (Sigma) and 1% P/S. The ovaries were freed from adding fat and punctured with a 26 1/2-gauge needle in a glass Petri dish to release granulosa cells and red blood cells. The remaining ovarian tissue was strained with medium using a 100 m cell strainer. The ovarian tissue in the cell strainer was placed in medium containing 4 mg/mL collagenase (Sigma (Merck KGaA, Darmstadt, Germany)), 40 μg/mL deoxyribonuclease I (Sigma), and 10 mg/mL BSA (RDT (Kent, UK)) and incubated for 1 h at 37 °C in an incubator after vortexing. The cells released by this digestion process were strained with a 40 μm cell strainer, centrifuged at 1000× *g* rpm for 5 min, and washed in medium three times to eliminate the remaining enzymes. Cell viability was assessed by the trypan blue exclusion assay. Finally, the cells were maintained at 37 °C in a humidified atmosphere containing 5% CO_2_. After the primary theca cells were stabilized, they were seeded at a density of 1.5 × 10^5^ cells/well in a 6-well plate. After 3 h of culture in a 6-well plate, we confirmed that the theca cells had attached to the plate and replaced the medium with medium containing 70 mM TAA or 100 μM etomoxir (Sigma) (* *p* < 0.05). After being cultured at 37 °C in an incubator for 24 h, the medium containing TAA or etomoxir was replaced with normal culture medium, and PD-MSCs (5 × 10^4^ cells/well) were cocultured in a plate with an 8 μm pore size insert (Falcon, New York, NY, USA) for 24 h. Then, the cells were collected.

### 2.4. RNA Extraction and Quantitative Real-Time PCR

Total RNA from frozen ovarian tissue was extracted using TRIzol reagent (Invitrogen, Waltham, MA, USA) according to the manufacturer’s instructions. RNA quality and concentration were verified by NanoDrop 2000 (Thermo Fisher Scientific, Waltham, MA, USA). Complementary DNA (cDNA) was synthesized using Superscript III reverse transcriptase with the following thermal steps: 65 °C for 5 min, 4 °C for 1 min, 50 °C for 1 h, and 72 °C for 15 min. Quantitative PCR was carried out using FS Universal SYBR Green Master ROX (Roche, Basel, Switzerland). The cycling parameters were 95 °C for 5 s, followed by 40 cycles of 95 °C for 5 s and 60 °C for 30 s. Expression levels were normalized to rat GAPDH, and each reaction was performed in triplicate. Primer sequences are listed in Table S1. The relative mRNA expression of the human gene was normalized by the cycle threshold value of human GAPDH.

### 2.5. Genomic DNA Extraction and mtDNA Copy Number Analysis

Genomic DNA was isolated from homogenized ovarian tissues using the QIAamp DNA Mini Kit (Qiagen, Valencia, CA, USA). DNA purity was determined spectrophotometrically, and 100 ng of gDNA per reaction was used for quantitative PCR with TaqMan Universal Master Mix (Applied Biosystems (Waltham, MA, USA)). The mitochondrial D-loop and nuclear β-actin genes were amplified to determine the mtDNA/nDNA ratio. Primer and probe sequences: D-loop: F: 5’-GGT TCT TAC TTC AGG GCC ATC A-3’, R: 5’-GAT TAG ACC CGT TAC CAT CGA GAT-3’, probe: JOE-TTG GTT CAT CGT CCA TAC GTT CCC CTT A-3’; rat β-actin: F: 5’-GGG ATG TTT GCT CCA ACC AA-3’, R: 5’-GCG CTT TTG ACT CAA GGA TTT AA-3’, probe: FAM-CGG TCG CCT TCA CCG TTC CAG TT-3’.

### 2.6. Western Blot Analysis

Ovarian tissues from each group of rats were lysed on ice in RIPA buffer (Sigma–Aldrich) supplemented with protease and phosphatase inhibitors (genDEPOT, Katy, TX, USA). Protein concentrations from individual rats were determined via a bicinchoninic acid assay (BCA) protein assay kit (Thermo Fisher Scientific). Equal concentrations of protein (10 μg) were separated by sodium dodecyl sulfate–polyacrylamide gel electrophoresis (SDS–PAGE) and transferred to polyvinylidene difluoride (PVDF) membranes (Bio–Rad Laboratories, Hercules, CA, USA). The membranes were then blocked with 5% BSA at room temperature for 1 h to prevent non-specific binding. After blocking, they were incubated with the primary antibody (diluted in 2% BSA) at 4 °C overnight. Finally, the membranes were incubated with the secondary antibody (diluted in 2% BSA) at RT for 1 h before signal detection. The primary antibodies are listed in Table S2. After incubation, the membranes were washed with 1X Tris-buffered saline-Tween 20 (TBS-T) and then incubated with the appropriate secondary antibody (diluted in 2% BSA) according to the host at RT for 1 h. After washing, the membranes were treated with reagents from the Clarity Western ECL Kit (Bio–Rad Laboratories) at RT for 5 min, and the protein bands were detected by a ChemiDoc XRS+ imaging system (Bio–Rad Laboratories). The bands were analyzed with ImageJ software (http://imagej.net/ij/, Wayne Rasband, Bethesda, MD, USA, accessed on 3 December 2025), and the fold change in intensity was determined as a measure of gene expression.

### 2.7. Hematoxylin and Eosin (H&E) Staining and Follicle Quantification

Fixed ovaries (10% neutral-buffered formalin) were embedded in paraffin and serially sectioned at 4 µm. After deparaffinization and rehydration, sections were stained with Harris’ hematoxylin and counterstained with eosin Y. Scanned images (3D HISTECH scanner, The Digital Pathology Company, Budapest, Hungary) were analyzed for the number of follicles at various developmental stages (primordial, primary, secondary, and antral) every 100 µm, following established counting criteria [45].

### 2.8. Immunofluorescence Staining

Frozen sections (7 µm) were fixed in 4% paraformaldehyde, blocked for 1 h, and incubated with primary antibodies overnight at 4 °C. After three washes with 1X PBS (5 min each), the tissues were incubated with secondary antibodies in diluent buffer at RT for 1 h. Sections were again washed three times with PBS and mounted with DAPI-containing mounting medium (Vectashield, Burlingame, CA, USA). Fluorescent images were captured using a fluorescence microscope, and representative areas from each sample were analyzed using ImageJ software (Wayne Rasband, Bethesda, MD, USA). The relative fluorescence intensity (fold change) was calculated as a measure of expression.

### 2.9. MitoTracker and MitoSOX Staining

Frozen ovarian tissue blocks were sectioned at a thickness of 7 μm and fixed with 4% PFA for 20 min. The sections were washed 3 times with 1X PBS at RT for 5 min each. To visualize mitochondrial distribution and ROS, as previously described, frozen sections were incubated with MitoTracker (mitochondria staining, green signals; Invitrogen Corporation, Waltham, MA, USA) at 50 nM and Mito SOX (superoxide staining, red signals; Invitrogen Corporation) at 1.5 μm, concentrations set according to the Invitrogen datasheet manuals, for 40 min at 37 °C. Slides were washed and mounted by using Vectashield antifade mounting medium with DAPI (Vector Labs, Burlingame, CA, USA). Confocal (Zeiss 780; Zeiss, Oberkochen, Germany) images were obtained at 20× magnification from random fields [46].

### 2.10. Immunohistochemical Staining

Deparaffinized ovarian sections underwent antigen retrieval in EDTA buffer (eLbio), endogenous peroxidase quenching (3% H_2_O_2_), and incubation with primary antibody at 4 °C overnight. After removing the unbound primary antibody, the tissue slides were incubated with Dako Real EnVision HRP Rabbit/Mouse secondary antibody (Dako, Carpinteria, CA, USA) at RT for 1 h. Detection was performed using the DAB substrate and counterstained with hematoxylin (Dako). After the reaction, the slides were rinsed with tap water and dehydrated. The slides were scanned with a 3D HISTECH.

### 2.11. TUNEL Assay

Deparaffinized ovarian sections were rinsed with TBS-T and stained using a TUNEL Assay Kit (Abcam, Waltham, MA, USA) according to the manufacturer’s instructions. Finally, the slides were counterstained with methyl green counterstain solution and mounted on a glass coverslip using organic mounting medium. To generate positive control, one or more slides were treated with 1 µg/µL DNase I in TBS/1 mM MgSO_4_ for 20 min at RT immediately following proteinase K treatment. The slides were scanned with a 3D HISTECH.

### 2.12. Nile Red Lipid Staining

Frozen ovarian sections (7 µm) were fixed with 4% PFA for 20 min. The sections were incubated with 0.5 µg/mL Nile red solution prepared from a 1 mg/mL acetone stock. After rinsing, the slides were mounted with DAPI-containing mounting medium (Vectashield, Burlingame, CA, USA). Lipid droplets were visualized under fluorescence microscopy. The primary outcomes were follicular development (stage-specific counts), mitochondrial function (mtDNA copy number and ATP), and ovarian lipid accumulation.

### 2.13. Enzyme-Linked Immunosorbent Assay

Blood was collected via the abdominal aorta, and serum was separated using Vacutainer tubes (Vacutainer; BD Biosciences, San Jose, CA, USA). Commercial ELISA kits were used to measure estrogen (E2; BioVision, Milpitas, CA, USA), anti-Mullerian hormone (AMH; Elabscience Biotechnology, Houston, TX, USA), follicle-stimulating hormone (FSH; Abnova, Taipei, Taiwan), testosterone (TES; CusaBio, Houston, TX, USA), and ATP (Thermo Fisher, Waltham, MA, USA) following the manufacturer’s instructions. Absorbance was determined using a microplate reader (BioTek Synergy, Winooski, VT, USA).

### 2.14. Statistical Analysis

All quantitative results are expressed as the mean ± standard deviation (SD). Group comparisons were made using Student’s *t*-test or one-way ANOVA with Tukey’s post hoc test (GraphPad Prism 5.0 Software, San Diego, CA, USA). A *p*-value < 0.05 was considered significant. Investigators analyzing data were blinded to group identity. For major outcomes, effect sizes and 95% confidence intervals are also reported.

## 3. Results

### 3.1. Alterations in Rats with TAA-Induced Injury

**Figure 1:**
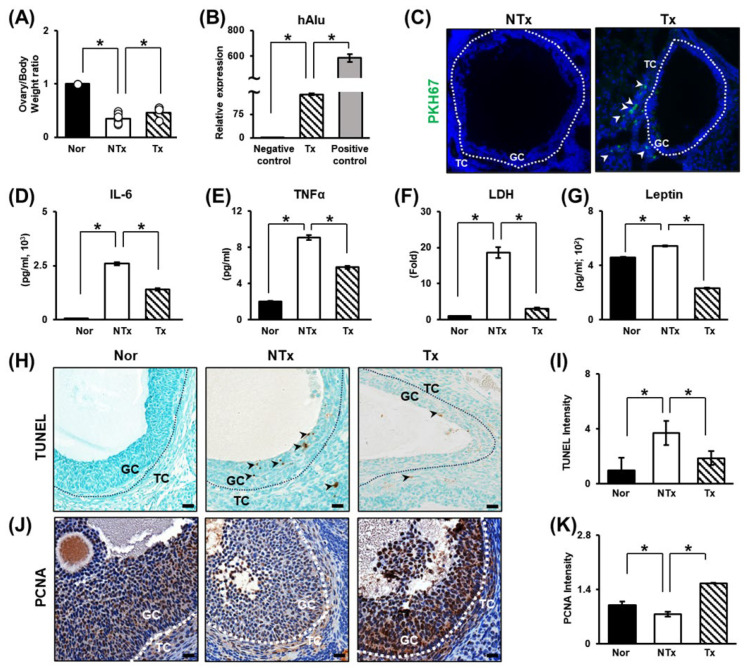
Increased alterations in the TAA-induced injury rat model and the engraftment capacity and therapeutic effects of PD-MSCs. (**A**) The ratio of ovarian weight to body weight after sacrifice. (**B**) The mRNA expression of hAlu in ovarian tissues determined by qRT–PCR. The mRNA expression level in PD-MSCs was used as a positive control. (**C**) PKH67-labeled PD-MSCs in ovarian tissues visualized by immunofluorescence. (**D**) The levels of IL-6 and (**E**) TNF-α in the serum determined by ELISA. (**F**) Serum LDH and (**G**) leptin levels were measured by ELISA. (**H**) The level and localization of DNA damage in follicles determined by TUNEL staining and (**I**) the quantification of the TUNEL staining intensity by 3D HISTECH 2.2 software. (**J**) Analysis of the expression and localization of PCNA in follicles by IHC staining and (**K**) the quantification of the PCNA staining intensity by 3D HISTECH software. Data means SD from three independent experiments: Normal *n* = 4, NTx *n* = 4, Tx *n* = 3. Statistics: one-way ANOVA with Tukey’s post hoc test. * *p* < 0.05.

Ovarian diameter and weight are known to affect ovarian function and oocyte quality [47]. The weight of the ovaries was significantly decreased in the NTx group compared to the normal group. In the Tx group, the body weight to ovary weight ratio was not quite normal, but a tendency toward recovery was observed (Figure 1A; * *p* < 0.05). Then, we aimed to confirm that PD-MSCs expressed the human Alu (hAlu) mRNA expression and that PKH67 fluorescence-labeled PD-MSCs were present in tissues and found that PD-MSCs were present and survived in the theca layer in the ovary in the Tx groups but not the NTx group (Figure 1B,C; * *p* < 0.05). These data indicate that transplanted PD-MSCs engrafted into the injured ovarian tissues in the TAA-treated rat model. Thus, it can be inferred that TAA has a direct negative effect on the ovaries and that PD-MSC transplantation may have a positive effect. TAA has been shown to cause injury to the liver through inflammation in high-sucrose-diet-fed animals [48]. To prove that TAA has a direct negative effect on the ovaries and that PD-MSC tended to alleviate this effect, the levels of pro-inflammatory factors and cell damage factors were analyzed. Inflammation caused by TAA injection was analyzed through blood chemistry tests and analysis of the levels of the pro-inflammatory factors interleukin-6 (IL-6) and tumor necrosis factor-α (TNFα). The levels of pro-inflammatory factors were dramatically increased in the NTx group, while inflammation was somewhat mitigated in the Tx group (Figure 1D,E; * *p* < 0.05). The level of cell damage was assessed by measuring lactate dehydrogenase (LDH) levels. LDH levels were dramatically increased in the NTx group but significantly decreased in the Tx group (Figure 1F; * *p* < 0.05). The level of leptin, which has recently emerged as a key link between the metabolic response and inflammation, was significantly increased in the NTx group and notably decreased sharply in the Tx group (Figure 1G; * *p* < 0.05). These results indicate that TAA affects the ovarian inflammatory response and cell damage and that PD-MSC transplantation can alleviate this effect.

It is known that apoptotic signals increase in response to metabolic disease and oxidative stress in the ovary [10,49,50]. Therefore, TUNEL staining was performed to demonstrate that TAA administration induces apoptosis in ovarian tissue. The TUNEL staining intensity was significantly increased in the NTx group, and the apoptotic signal was significantly decreased in the Tx group compared to the NTx group (Figure 1H,I; * *p* < 0.05). Furthermore, immunohistochemistry for PCNA, a proliferation factor, was performed. While the apoptotic signal intensity was significantly increased in the NTx group, it was low. In contrast, the expression of this proliferation factor was outstandingly increased in the Tx group (Figure 1J,K; * *p* < 0.05). The data show that TAA induces apoptosis in granulosa cells in ovarian tissue. After PD-MSC transplantation, apoptosis was decreased, and the expression of the proliferation factor was increased. In addition, mature follicles were protected. Thus, PD-MSC transplantation can inhibit damage. The data suggests that PD-MSCs inhibit ovarian follicle cell apoptosis induced by TAA injection and exert therapeutic effects by enhancing proliferation.

### 3.2. Therapeutic Effects on Follicular Development and Sex Hormone Levels

**Figure 2:**
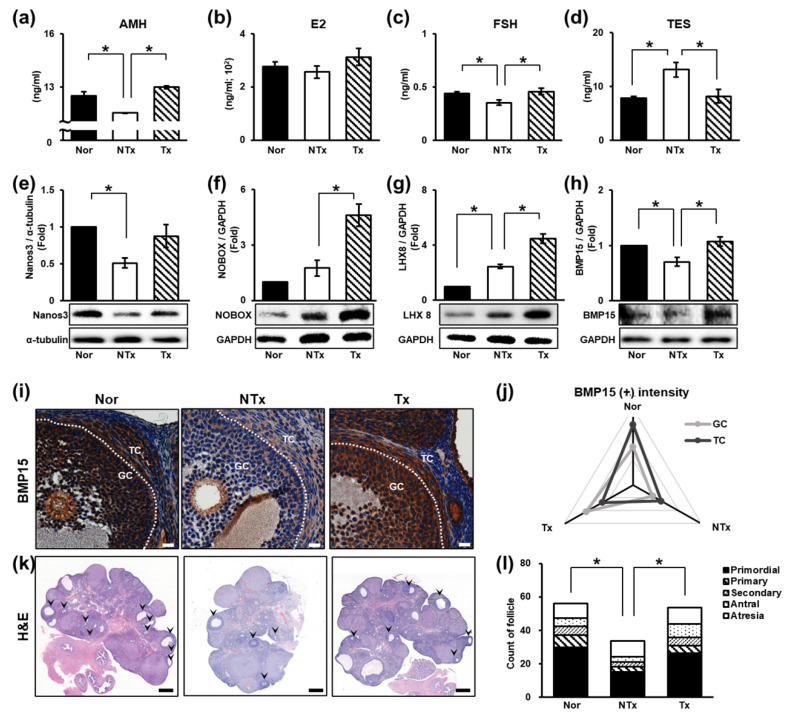
Therapeutic effect of PD-MSCs on follicular development. (**a**) The levels of AMH, (**b**) E2, (**c**) FSH, and (**d**) TES in individual serum samples determined by ELISA. (**e**–**h**) The expression of proteins related to follicular development determined by Western blot analysis. (**i**) The expression and localization of BMP15 determined by immunohistochemistry staining and (**j**) quantified by 3D HISTECH software. (**k**) Histological analysis and (**l**) the quantification of follicle number by H&E staining and 3D HISTECH software. Data means SD from three independent experiments: Normal *n* = 4, NTx *n* = 4, and Tx *n* = 3. Statistics: one-way ANOVA with Tukey’s post hoc test. * *p* < 0.05.

Follicular development refers to the growth and maturation of follicles within the ovary and is one of the major indicators of ovarian function [51,52]. To observe changes in ovarian function, the levels of sex hormones (e.g., AMH, E2, FSH, and TES) in the blood were determined by ELISA. AMH and E2 levels significantly decreased in the NTx group and significantly increased in the Tx group. In addition, the level of FSH showed the same trend. In contrast, the TES level increased in the NTx group and decreased in the Tx group (Figure 2a–d; * *p* < 0.05).

The levels of follicular development factors [e.g., Nanos3, NOBOX, bone morphogenetic protein 15 (BMP15), and LIM homeobox 8 (LHX8)] were measured. The protein expression of Nanos3, which affects early follicular development, was significantly decreased in the NTx group compared to the normal group and increased in the Tx group compared to the NTx group (Figure 2e; * *p* < 0.05). The protein expression of NOBOX, which plays an important role in specifying the expression patterns of oocyte-specific genes essential for follicular development, showed low expression in the normal group but was significantly increased in the Tx group compared to the NTx group (Figure 2f; * *p* < 0.05). The protein expression of Lhx8, which regulates primordial follicles activation, was notably increased in the Tx group compared to the NTx group (Figure 2g; * *p* < 0.05). The protein expression of BMP15, which is involved in follicle maturation, was significantly decreased in the NTx group compared to the normal group and remarkably increased in the Tx group compared to the NTx group (Figure 2h; * *p* < 0.05). Then, we confirmed the localization and gene expression of BMP15 in ovarian tissues of TAA-injured rats (Figure 2i). The gene expression of BMP15 decreased in granulosa cells and theca cells in the NTx group compared to the normal group. The intensity of BMP15 was increased in the Tx group similar to the normal group (Figure 2j; * *p* < 0.05).

To confirm the effect of PD-MSCs on follicular development, we performed H&E staining using serially sectioned ovarian tissues (Figure 2k). In the NTx group, the activation of primordial follicles was inhibited, whereas in the Tx group, the activation of primordial follicles occurs as in the normal group. The mature follicle count (e.g., secondary follicles and antral follicles) was significantly decreased, and atretic follicles were increased, whereas in the Tx group, a tendency toward protection of mature ovarian follicles was observed (Figure 2l, Table 1; * and ** *p* < 0.05).

Our data show that TAA inhibits follicular development and affects sex hormone levels. However, it is known that when E2 levels decrease, the FSH level increases when ovarian function is impaired [53]. In PCOS, the FSH level is known to remain the same or decrease, and it is inferred that the FSH level decreases due to metabolic dysfunction [54]. PD-MSC transplantation has therapeutic potential, such as the ability to improve follicular development, restore sex hormone levels, and protect mature follicles.

### 3.3. Changes in Mitochondrial Function in the Ovaries

**Figure 3:**
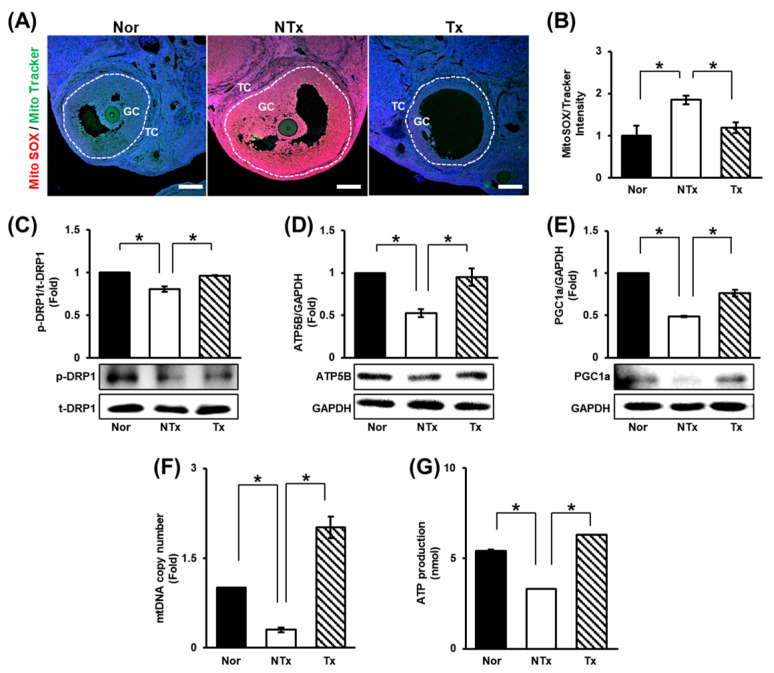
Effect of PD-MSCs on mitochondrial function. (**A**) Analysis of the expression and localization of ROS (MitoSOX) and mitochondria (MitoTracker) in follicles by immunofluorescence staining and (**B**) the quantification of the staining intensity (the ratio of MitoSOX to MitoTracker) by ImageJ software. (**C**–**E**) The expression of proteins related to mitochondrial dynamics and mitochondrial biogenesis was determined by Western blot. (**F**) The mtDNA copy number in gDNA determined by the TaqMan assay. (**G**) Analysis of ATP production in protein lysates by ELISA. Data means SD from three independent experiments: Normal *n* = 4, NTx *n* = 4, and Tx *n* = 3. Statistics: one-way ANOVA with Tukey’s post hoc test. * *p* < 0.05.

In a previous study, TAA injection was shown to affect liver and ovarian function through oxidative stress [28]. In addition to oxidative stress, mitochondrial dysfunction is known to cause ovarian dysfunction [55,56,57,58]. We assessed the ratio of MitoSOX to MitoTracker staining and found that the ROS level in ovarian tissues dramatically increased in the NTx group compared to the normal group and significantly decreased in the Tx group compared to the NTx group (Figure 3A,B; * *p* < 0.05).

Furthermore, the levels of mitochondrial dynamics factors [e.g., dynamin-related protein 1 (DRP1)] were analyzed. The expression of DRP1, which is involved in mitochondrial fission, was decreased in the NTx group and increased in the Tx group (Figure 3C; * *p* < 0.05). Subsequently, the levels of factors involved in mitochondrial biogenesis [e.g., peroxisome ATP synthase F1 subunit beta (ATP5B), proliferator-activated receptor-gamma coactivator-1alpha (PGC1a)] decreased in the NTx group and increased in the Tx group (Figure 3D,E; * *p* < 0.05).

To determine the changes in the number and function of mitochondria through mitochondrial dynamics, the mtDNA copy number and ATP production were analyzed. The results showed that both the mtDNA copy number and ATP production were decreased in the NTx group and increased in the Tx group (Figure 3G,H; * *p* < 0.05). Based on these data, TAA increases the ROS level in the ovaries, and PD-MSCs decrease it. In addition, PD-MSCs were confirmed to induce mitochondrial fission and mitochondrial biogenesis. Analysis of the mtDNA copy number revealed that TAA reduced the number of mitochondria. Additionally, analysis of ATP production showed that mitochondrial function was impaired by TAA injection. However, PD-MSC transplantation improved the number and function of mitochondria. Thus, TAA induces oxidative stress and mitochondrial dysfunction in ovarian tissues, and PD-MSC transplantation seems to alleviate these changes.

### 3.4. Analysis of Lipid Metabolism in the Ovaries

**Figure 4:**
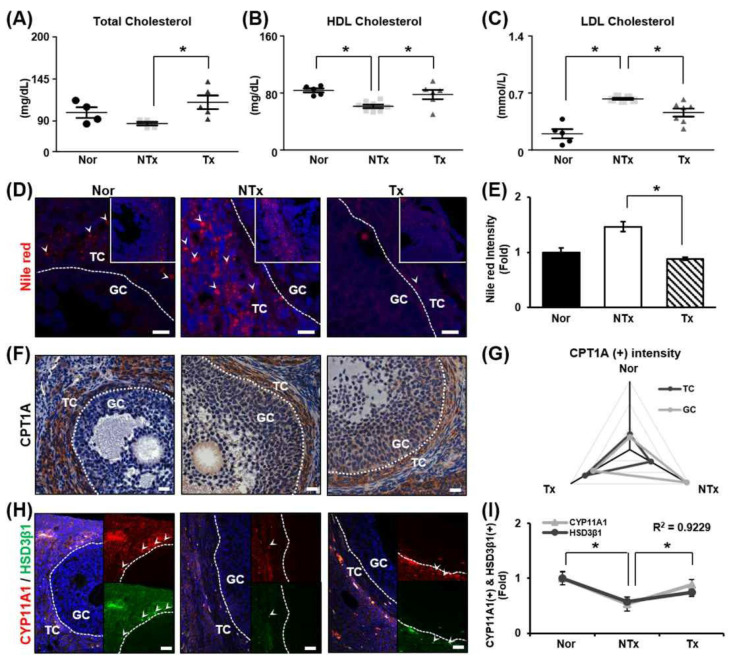
Effect of PD-MSCs on lipid metabolism and steroidogenesis in the ovaries. (**A**) The levels of total cholesterol, (**B**) HDL, and (**C**) LDL in the serum determined by a serological assay. (**D**) Staining of lipid droplets by Nile red staining. (**E**) Quantification of the staining intensity by ImageJ software. (**F**) Analysis of the expression and localization of CPT1A in follicles by IHC staining. (**G**) Quantification of the CPT1A staining intensity in follicles by 3D HISTECH software. (**H**) Analysis of the expression and localization of CYP11A1 and HSD3β1 in follicles by immunofluorescence staining and (**I**) quantification of the CYP11A1 and HSD3β1 staining intensity by ImageJ software. Data means SD from three independent experiments: Normal *n* = 4, NTx *n* = 4, Tx *n* = 3. Statistics: one-way ANOVA with Tukey’s post hoc test. * *p* < 0.05.

TAA was shown to induce lipid accumulation in the liver [29]. It has been reported that lipids accumulate in the context of metabolic diseases, and mitochondrial abnormality ties in the ovaries [59,60]. Accordingly, the change in blood cholesterol level was analyzed to assess the accumulation of lipids in the ovaries induced by TAA. Total cholesterol and HDL levels were decreased, and LDL levels were notably increased in the NTx group, whereas total cholesterol and HDL levels were significantly increased and LDL levels were remarkably decreased in the Tx group compared to the NTx group (Figure 4A–C; * *p* < 0.05). An increase in cholesterol levels in blood samples confirmed that lipids had accumulated in the ovaries.

Analysis of lipid accumulation in the ovaries using Nile red staining revealed that lipid droplets accumulated in the theca cell layer. Lipid droplets were found to increase dramatically in the NTx group and decrease in the Tx group (Figure 4D,E; * *p* < 0.05). CPT1A, a lipid metabolism marker, was mainly observed in the theca cell layer. In the NTx group, CPT1A expression was higher in granulosa cells than in theca cells. However, in the Tx group, the pattern of CPT1A expression was similar to that in the normal group (Figure 4F,G; * *p* < 0.05). Additionally, the levels of the steroidogenesis markers (e.g., CYP11A1 and HSD3β1) were decreased in the NTx group compared to the normal group and increased in the Tx group compared to the NTx group. The gene expression of CYP11A1 was similar to the gene expression of HSD3β1 in ovarian tissue, and the correlation of the R value between CYP11A1 expression and HSD3β1 expression was 0.9229, confirming a correlation (Figure 4H,I; * *p* < 0.05). According to the results, TAA caused lipid accumulation in the ovaries, which posed an obstacle to lipid metabolism and steroidogenesis. It was confirmed that PD-MSC transplantation exerted a therapeutic effect on lipid metabolism and steroidogenesis in the ovaries.

### 3.5. Effect of Coculture with PD-MSCs on Lipid Metabolism and Steroidogenesis in CPT1A Inhibitor-Treated Primary Theca Cells (In Vitro)

**Figure 5:**
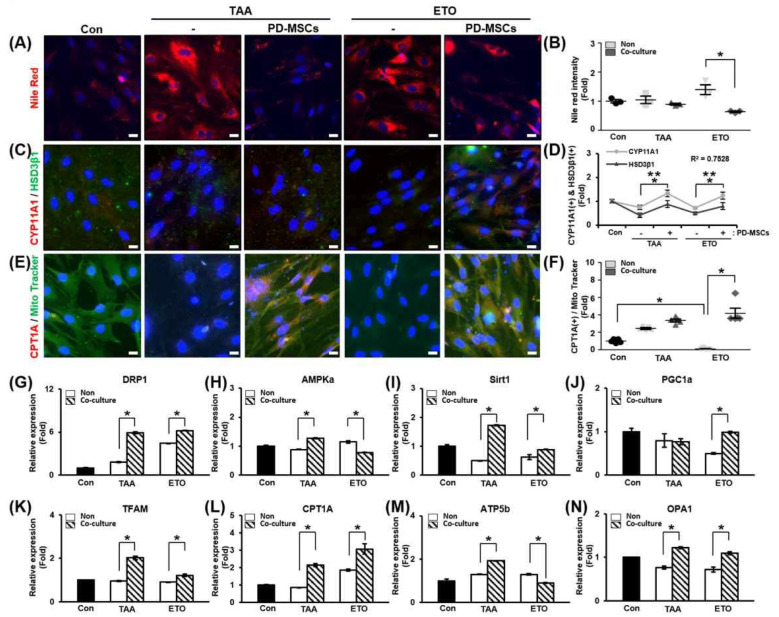
Effect of PD-MSCs on lipid metabolism and steroidogenesis in primary theca cells. (**A**) The intensity of Nile red staining and (**B**) histological analysis of primary theca cells by immunofluorescence and ImageJ software. (**C**) The expression levels of CYP11A1 and HSD3β1, which are related to steroidogenesis, in primary theca cells were determined by immunofluorescence and (**D**) quantified by ImageJ software. *: significant difference (*p* < 0.05) in CYP11A1 expression; **: significant difference (*p* < 0.05) in HSD3β1 expression. (**E**) The expression levels of CPT1A, which is related to lipid metabolism, and the abundance of mitochondria (MitoTracker) in primary theca cells were determined by immunofluorescence, and (**F**) quantified by ImageJ software. (**G**–**N**) The mRNA expression of mitochondrial dynamics and biogenesis markers in primary theca cells determined by qRT-PCR. Data means SD from three independent experiments: Normal *n* = 4, NTx *n* = 4, Tx *n* = 3. Statistics: one-way ANOVA with Tukey’s post hoc test. * *p* < 0.05.

In previous experiments, lipid accumulation and metabolism in ovaries were found to mainly occur in theca cells under normal conditions. Therefore, primary theca cells were isolated from rat ovaries, and the cells were confirmed to be theca cells. Lipid accumulation of theca cells was confirmed by Nile red staining after theca cells were treated with TAA or etomoxir, an inhibitor of CPT1A, for induction of lipid toxicity. As a result, lipid accumulation was alleviated in TAA- or etomoxir-treated theca cells cocultured with PD-MSCs compared to TAA- or etomoxir-treated theca cells alone. In particular, lipid accumulation was significantly decreased in etomoxir-treated theca cells cocultured with PD-MSCs compared to etomoxir-treated theca cells (Figure 5A,B; * *p* < 0.05).

To confirm that steroidogenesis occurred in theca cells, we analyzed the colocalization and gene expression of steroidogenesis markers (e.g., CYP11A1 and HSD3β1) (Figure 5C). The gene expression levels of CYP11A1 and HSD3β1 were also increased in the coculture groups compared to the non-coculture groups. The relationship between CYP11A1 and HSD3β1 gene expression was analyzed in TAA- or etomoxir-treated theca cells. The correlation of R value between CYP11A1 expression and HSD3β1 expression was 0.7528 (Figure 5D; * and ** *p* < 0.05). This data demonstrates that PD-MSCs activate steroidogenesis in theca cells induced by lipotoxicity.

To evaluate that FAO was regulated in theca cells, we analyzed the localization and gene expression of CPT1A (Figure 5E). The expression of CPT1A was significantly decreased in the etomoxir-treated group compared to the control group. The CPT1A staining intensity was markedly increased in etomoxir-treated theca cells cocultured with PD-MSCs compared to etomoxir-treated theca cells (Figure 5F; * *p* < 0.05). These results suggest that PD-MSCs regulate FAO in theca cells by activating the expression of CPT1A.

Additionally, we confirmed the mRNA expression of mitochondrial biogenesis and dynamic markers in TAA- or etomoxir-treated theca cells. The mRNA expression of DRP1, which is involved in mitochondrial fission, was increased in the coculture groups compared to the non-coculture groups (Figure 5G; * *p* < 0.05). In addition, the mRNA expression of AMP-activated protein kinase alpha (AMPKa), Sirt1 and PGC1a, and mitochondrial transcription factor A (TFAM), which are involved in mitochondrial biogenesis, increased in the coculture group, and accordingly, the mRNA expression of CPT1A was increased in the coculture group (Figure 5H,I; * *p* < 0.05). The mRNA expression of ATP5b, which is involved in ATP production, increased in TAA-treated theca cells cocultured with PD-MSCs compared to non-cocultured TAA-treated theca cells, but this was not the case for etomoxir-treated theca cells cocultured with PD-MSCs (Figure 5M; * *p* < 0.05). The mRNA expression of OPA1, which is involved in mitochondrial fusion, was slightly increased in the coculture groups compared to the non-coculture groups (Figure 5G; * *p* < 0.05). Based on these data, PD-MSCs can influence lipid metabolism, steroidogenesis, and mitochondrial function in theca cells. Thus, PD-MSCs exert a therapeutic effect in vitro as well as in vivo.

## 4. Discussion

Lipids are efficient sources of energy reserves and glycogen and are physiologically active substances that support cell survival and regulate hormones. In particular, circulating lipids (e.g., LDL and HDL) have been used as diagnostic markers for various diseases, and increasing evidence for their associations with reproductive diseases was recently obtained [61]. Mumford and colleagues reported that the level of cholesterol is associated with endogenous estrogen levels throughout the menstrual cycle. The level of LDL decreases in the luteal phase, and the level of HDL increases during ovulation [62,63]. Estradiol, a major hormone involved in reproductive development, is associated with higher levels of HDL in the follicular phase of the menstrual cycle in menstruating women [64]. However, excessive lipid accumulation and abnormal lipid metabolism adversely affect ovarian tissue. Abnormal accumulation of lipids in the ovaries leads to the release of high doses of leptin from adipocytes. High levels of leptin block follicular development and further induce oxidative stress due to lipotoxicity through lipid accumulation [65]. Hence, we constructed a lipid metabolic disease model using TAA, a substance that induces liver disease through abnormal lipid metabolism, to examine the relationship between lipid levels and ovarian function. Blood chemistry tests showed that HDL levels were increased and LDL decreased in the serum in TAA-induced injury model rats after PD-MSC transplantation. Additionally, it was observed that TAA administration induced abnormal lipid accumulation in ovarian tissue. Interestingly, CPT1A by transplanted PD-MSC decreased lipid accumulation in ovarian tissues in the TAA-induced injury rat model and activated mitochondria.

Mitochondria, regulated by lipid metabolism, play a crucial role in regulating ovarian function. Itami and their colleagues demonstrated that palmitic acid induces lipid accumulation in oocytes via mitochondrial protein hyperacetylation and dysfunction. Therefore, excessive lipid accumulation affects the expression of mitochondrial biogenesis markers such as AMPK and Sirt3 in oocytes and impairs mitochondrial function, causing ovarian dysfunction [66]. In addition, metabolic ovarian dysfunction is alleviated by the clearance of excessive accumulated lipids [59]. CPT1A is known to play a crucial role in mitochondrial functions, such as ATP production and steroidogenesis, by facilitating lipid metabolism within the mitochondria. CPT1A follows this process by accepting cholesterol at the outer mitochondrial membrane and initiating beta-oxidation [23]. Rua and their colleagues demonstrated that CPT1A expression was increased in a high-fat diet rat model. They proposed CPT1A as an indicator of increased risk for metabolic disorders [67]. Cheshmeh and their colleagues reported that CPT1A shows high expression in women with PCOS, but its expression decreases after following a low-calorie diet [68]. While there are reports that activation of CPT1A induces lipid accumulation, there are also reports suggesting that activation of CPT1A improves mitochondrial dynamics and steroidogenesis by regulating lipid metabolism, suggesting therapeutic potential. According to Soler-Vázquez and their colleagues, in obese mice transplanted with adipose-derived mesenchymal stem cells (AD-MSCs) expressing CPT1A, hyperglycemia decreased, and obesity improved [69]. Calderon-Dominguez and their colleagues demonstrated that activated CPT1A improves the reduction in brown adipocytes by increasing FAO and mitochondrial activity [70].

However, studies elucidating the regenerative mechanisms of activated CPT1A in lipid metabolism and mitochondrial dynamics, as well as how MSCs modulate mitochondrial dynamics, remain limited. In this study, we investigated the therapeutic mechanisms linking lipid metabolism and mitochondrial dynamics mediated by CPT1A activation. Interestingly, we found that TAA impaired mitochondrial function in the ovaries, as indicated by decreases in the mtDNA copy number and ATP production in the ovaries, and Nile red staining confirmed that lipids accumulated in the theca cell layer of follicles in the presence of CPT1A. These results indicate that CPT1A activated by transplanted PD-MSCs acts on the theca cell layer in ovarian follicles to improve mitochondrial function in the ovaries and prevent the accumulation of lipids. In addition, the relatively small sample size of the in vivo experiments and reliance on a single TAA-induced model represent important limitations that may affect the generalizability of our findings. Therefore, future studies using larger cohorts and diverse metabolic dysfunction models are warranted to further validate these mechanisms.

It has been reported that metabolism and ATP levels in cumulus cells, which are close to oocytes in mature follicles, are correlated with the quality of oocytes and healthy embryogenic development [71]. In addition, it is known that the composition of fatty acids in follicular fluid is affected by dietary fat as well as obesity and has a significant effect on follicular development according to the content of saturated fatty acids versus unsaturated fatty acids in an in vitro maturation system [72]. Fatty acids generated from adipose tissue breakdown are further metabolized through mitochondrial beta-oxidation to produce ATP, and pharmacological inhibition of beta-oxidation inhibits oocyte maturation and embryonic development [73,74]. In particular, it has been reported that an abnormal mtDNA copy number and mitochondrial dysfunction are major risk factors for PCOS [56]. Hence, we verified the change in mitochondrial function in TAA-injured ovarian tissues and then investigated the correlation between these changes and beta-oxidation in vitro. Our data indicated that transplanted PD-MSCs restored mitochondrial function, including by alleviating oxidative stress induced by TAA. As shown in Figure 3, PD-MSC transplantation decreased ROS levels and increased the mtDNA copy number and ATP production, which are related to mitochondrial function. TAA and etomoxir were administered to the theca cells of follicles that first accepted lipids to investigate the effect of TAA on beta-oxidation. As shown in Figure 5, TAA inhibited beta-oxidation, similar to etomoxir, thereby increasing lipid accumulation and reducing the expression of genes related to steroidogenesis in theca cells. We also found that transplanted PD-MSCs induced steroidogenesis by activating beta-oxidation as well as reducing lipid accumulation.

Several researchers have reported that stem cells, which have higher self-renewal potential and multi-differentiation potential, exert immunomodulatory effects and have therapeutic potential for various diseases that specifically affect females, including perimenopause, PCOS, and infertility, through ovarian dysfunction [75]. In particular, studies on the therapeutic effect of metformin and selenium on PCOS have been conducted. It has been reported that these treatments restore oxidative stress and lipid levels to normal and, as in our study, stabilize hormone levels. However, since selenium can only stay in the body for 24 h, periodic administration is necessary; in contrast, because it has a long-acting effect, MSC transplantation is considered to have a more favorable therapeutic effect. MSCs have been shown in numerous preclinical studies to improve follicular development in PCOS through modes of action including anti-inflammatory effects, antioxidant activity, and regulation of lipid metabolism, and clinical studies are currently underway [76]. When MSC-based therapies are considered for clinical use in PCOS patients, the safety and stability of the MSCs must be ensured. According to Yoon, the production of clinical-grade MSCs requires the expansion of large numbers of cells in vitro, but extensive passaging can lead to cellular transformation and may increase the risk of tumorigenicity [77]. Therefore, careful consideration of the optimal MSC dose and administration timing is critical for safe and effective therapy.

In summary, previous studies have confirmed that TAA leads to dysfunction of the ovaries by increasing oxidative stress and abnormal lipid accumulation. Additionally, we demonstrated that transplanted PD-MSCs helped restore ovarian function by inhibiting lipid accumulation through activation of beta-oxidation. Therefore, we investigated whether PD-MSCs can regulate lipid metabolism, and the findings suggest that PD-MSC transplantation may ameliorate abnormal lipid metabolism to improve reproductive function. Therefore, these findings offer new insights into the therapeutic potentiation of stem cell therapies for reproductive diseases and provide new avenues for the development of more efficient therapies in the field of regenerative medicine.

## 5. Conclusions

In conclusion, TAA leads to mitochondrial dysfunction through inhibition of CPT1A expression, including by inducing oxidative stress in the ovaries, resulting in symptoms similar to early menopause in women. Accordingly, PD-MSC transplantation improves mitochondrial function by inducing the gene expression of CPT1A and relieving oxidative stress, thereby restoring ovarian function (graphical abstract). This study provides insights for stem cell therapies and shows the therapeutic potential of regenerative medicine-based treatments for reproductive diseases.

## Figures and Tables

**Table 1:** The ratio of follicle counting in the TAA-injured rat model.

	Primordial	Primary	Secondary	Antral	Atresia
Normal (*n* = 4)	29.67 ± 4.42	7.58 ± 1.44	5.01 ± 1.46	5.13 ± 0.31	8.50 ± 1.31
NTx (*n* = 4)	15.09 ± 0.54 *	3.15 ± 0.29 *	2.77 ± 0.46	3.26 ± 0.47 *	9.24 ± 0.40
Tx (*n* = 3)	26.15 ± 3.80 **	4.79 ± 0.75	4.89 ± 0.67 **	8.10 ± 0.81 **	9.80 ± 0.77

*: NTx vs. Nor (*p* < 0.05); **: Tx vs. NTx (*p* < 0.05).

## Data Availability

The data supporting the findings of this study are available from the corresponding author upon reasonable request.

## Supplementary Materials

The following supporting information can be downloaded at: https://www.mdpi.com/article/10.3390/cells14241932/s1. Table S1: A list of primer used for Quantitative real time polymerase chain reaction; Table S2: A list of anti-body used for Western blot and immunohistochemistry staining and immunofluorescence staining.
