# Supplementary material for: Placental-Derived Mesenchymal Stem Cells Triggers Lipid Metabolism in a Rat Model Thioacetamide-Induced Ovarian Disease via Increased CPT1A Expression for Mitochondrial Dynamics
Source: Cells. 2025 Dec 5;14(24):1932. doi: 10.3390/cells14241932 (PMC12731433; doi:10.3390/cells14241932)
Supplement: Supplementary file 1 [file cells-14-01932-s001.zip › cells-3967639-supplementary.pdf]

Supplementary Table S1. A list of primer used for Quantitative real time polymerase chain reaction.

	Gene	Primer	Tm (°C)	NCBI ref.
qRT-PCP	**Rat *CYP17A1***	F: 5’-CATGCTGGAGGCCACTATCC-3’<br>R: 5’-AGTGCCCAGAGATTGACGAC-3’	60	NM_012753.3
	**Rat *DRP1***	F: 5’-GACTTTGCTGATGCCTGTGG-3’<br>R: 5’-GTTGCCTGTTGTCGGTTCC-3’	58	XM_039087901.1
	**Rat *AMPKa***	F: 5’-GGGATCCATCAGCAACTATCG-3’<br>R: 5’-GGGAGGTCACGGATGAGG-3’	58	NM_019142.3
	**Rat *Sirt1***	F: 5’-TATGCTCGCCTTGCTGTGGA-3’<br>R: 5’-GCTGAGTTGCTGGATTTTGTGT-3’	59	NM_001372090.1
	**Rat *PGC1a***	F: 5’-GCACACATCGCAATTCTCCC-3’<br>R: 5’-CTCTGCGGTATTCGTCCCTC-3’	59	NM_031347.1
	**Rat *TFAM***	F: 5’-CGCCTAAAGAAGAAAGCACA-3’<br>R: 5’-GCCCAACTTCAGCCATTT-3’	55	NM_031326.2
	**Rat *CPT1A***	F: 5’-CCGAGAAGGGAGGACAGAGA-3’<br>R: 5’-GTACAGGTGCTGGTGCTTCT-3’	59	XM_039102321.1
	**Rat *ATP5b***	F: 5’-GGTCACATGGGAAAGCTGGT-3’<br>R: 5’-TGTAGAAGGCTTGTTCCGGG-3’	58	NM_134364.1
	**Rat *Opa1***	F: 5’-TCTTCACTGCGGGTACACCT-3’<br>R: 5’-TCCTTCTCCAAACGCTCCAG-3’	59	NM_133585.3
	**Rat *GAPDH***	F: 5'-TCCCTCAAGATTGTCAGCAA-3'<br>R: 5'-AGATCCACAACGGATACATT-3'	55	NM_017008.4
	**Human *CYP17A1***	F: 5’-TTTCAGCCGCACACCAACTA-3’<br>R: 5’-AGCGCCCACAGATTGATGAT-3’	60	NM_000102.4
	**Human *Alu***	F: 5’-GGAGGCTGAGGCAGGAGAA-3’<br>R: 5’-ATCTCGGCTCACTGCAACCT-3’	55	NM_002715
	**Human *GAPDH***	F: 5’-CGAGATCCCTCCAAAATCAA-3’<br>R: 5’-TGTGGTCATGAGTCCTTCCA-3’	55	NM_001357943.2

Supplementary Table S2. A list of anti-body used for western blotting and immunohistochemistry staining and immunofluorescence staining.

Species	Antibody	Company
**Mouse**	NOBOX	Santacruz
	ATP5B	Santacruz
	α-tubulin	CALBIOCHEM
	HSD3β1	Novus
	CPT1A	Abcam
	HRP-linked antibody (Ms)	Cell signaling
**Rabbit**	Nanos3	Abcam
	BMP 15	Mybiosource
	LHX 8	Abcam
	OPA1	Abcam
	p-DRP1	Invitrogen
	t-DRP1	Cell signaling
	PGC1a	Novus
	GAPDH	Abfrontier
	CYP11A1	Cell signaling
	PCNA	Santacruz
	HRP-linked antibody (Rb)	Cell signaling
